# (5*E*)-Dimethyl 2-bromo­methyl-5-cyclo­hexyl­imino-2-phenyl-2,5-dihydro­furan-3,4-dicarboxyl­ate

**DOI:** 10.1107/S1600536809008496

**Published:** 2009-03-14

**Authors:** Afsaneh Zonouzi, Mojtaba Biniaz, Hossein Rahmani, Seik Weng Ng

**Affiliations:** a Department of Chemistry, College of Science, University of Tehran, PO Box 13145-143, Tehran, Iran; b Institute of Chemical Industries, Iranian Research Organization for Science and Technology, PO Box 15815-358, Tehran, Iran; c Department of Chemistry, University of Malaya, 50603 Kuala Lumpur, Malaysia

## Abstract

The mol­ecule of the title compound, C_21_H_24_BrNO_5_, has a planar furan ring [maximum deviation = 0.025 (3) Å]. The carboxy­methyl group in the 3-position is nearly coplanar with this ring [dihedral angle = 7.9 (1)°], whereas that in the 4-position is nearly perpendicular to it [dihedral angle = 78.9 (1) Å].

## Related literature

The imino­lactone was synthesized by the one-pot, solvent-free reaction of dimethyl acetyl­enedicarboxyl­ate, cyclo­hexyl isocyanide and α-bromo­acetophenone under microwave irradiation; for other synthetic methods, see: Ma & Xie (2002 ▶, 2005 ▶); Nair *et al.* (2000 ▶); Villemin & Liao (2003 ▶).
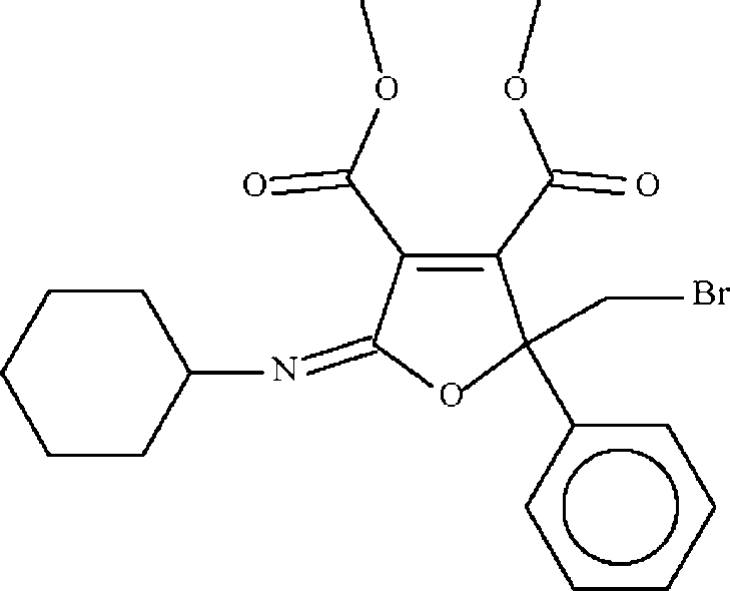


## Experimental

### 

#### Crystal data

C_21_H_24_BrNO_5_
*M* *_r_* = 450.32
Monoclinic, 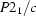

*a* = 16.8599 (3) Å
*b* = 7.2871 (1) Å
*c* = 17.4145 (3) Å
β = 97.330 (1)°
*V* = 2122.06 (6) Å^3^
*Z* = 4
Mo *K*α radiation
μ = 1.97 mm^−1^
*T* = 123 K
0.30 × 0.15 × 0.10 mm

#### Data collection

Bruker SMART APEX diffractometer
Absorption correction: multi-scan (*SADABS*; Sheldrick, 1996 ▶) *T*_min_ = 0.590, *T*_max_ = 0.828
19142 measured reflections
4873 independent reflections
4034 reflections with *I* > 2σ(*I*)
*R*_int_ = 0.046

#### Refinement

*R*[*F* ^2^ > 2σ(*F* ^2^)] = 0.053
*wR*(*F* ^2^) = 0.156
*S* = 1.08
4873 reflections
255 parameters
H-atom parameters constrained
Δρ_max_ = 2.31 e Å^−3^
Δρ_min_ = −0.67 e Å^−3^

### 

Data collection: *APEX2* (Bruker, 2008 ▶); cell refinement: *SAINT* (Bruker, 2008 ▶); data reduction: *SAINT*; program(s) used to solve structure: *SHELXS97* (Sheldrick, 2008 ▶); program(s) used to refine structure: *SHELXL97* (Sheldrick, 2008 ▶); molecular graphics: *X-SEED* (Barbour, 2001 ▶); software used to prepare material for publication: *publCIF* (Westrip, 2009 ▶).

## Supplementary Material

Crystal structure: contains datablocks global, I. DOI: 10.1107/S1600536809008496/tk2389sup1.cif

Structure factors: contains datablocks I. DOI: 10.1107/S1600536809008496/tk2389Isup2.hkl

Additional supplementary materials:  crystallographic information; 3D view; checkCIF report

## Figures and Tables

**Fig. 1.**
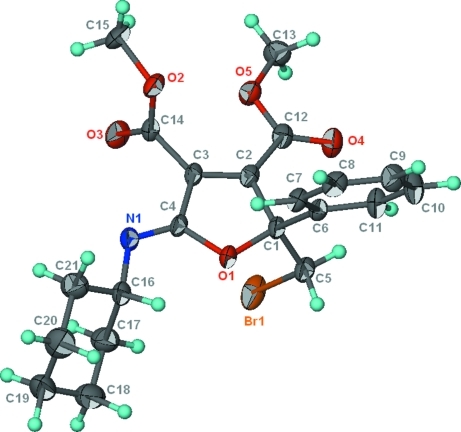
Thermal ellisoid plot (Barbour, 2001) of C21H24BrNO5; probability levels are set at 70% and H-atoms are drawn as spheres of arbitrary radius.

## supplementary crystallographic information

### Experimental

To a mixture of 2-bromo-1-phenyl ethanone (α-bromo acetophenone, 0.398 g, 2 mmol) and dimethyl acetylenedicarboxylate (0.25 ml, 2 mmol), cyclohexyl isocyanide (0.25 ml, 2 mmol) was added. Irradiation of the mixture with microwave radiation (180 W) for 5 min produced the title iminolactone. The reaction was monitored by TLC (ethyl acetate *n*-hexane 4:1) until no α-bromoacetophenone was detectable. The product was recrystalized from methanol; yield 90%, m.p. 351 K.

### Refinement

Carbon-bound H-atoms were placed in calculated positions (C–H 0.95 to 0.99 Å) and were included in the refinement in the riding model approximation, with *U*(H) set to 1.2 to 1.5U_eq_(C).

The final difference Fourier map had a large peak/deep hole in the vicinity of the bromide atom. Attempts to model the bromide atom as being disordered over two positions did not lead to any improvement in the refinement.

### Crystal data

**Table:**

C_21_H_24_BrNO_5_	*F*(000) = 928
*M**_r_* = 450.32	*D*_x_ = 1.410 Mg m^−^^3^
Monoclinic, *P*2_1_/*c*	Mo *K*α radiation, λ = 0.71073 Å
Hall symbol: -P 2ybc	Cell parameters from 3661 reflections
*a* = 16.8599 (3) Å	θ = 2.7–28.3°
*b* = 7.2871 (1) Å	µ = 1.97 mm^−^^1^
*c* = 17.4145 (3) Å	*T* = 123 K
β = 97.330 (1)°	Prism, colorless
*V* = 2122.06 (6) Å^3^	0.30 × 0.15 × 0.10 mm
*Z* = 4	

### Data collection

**Table:**

Bruker SMART APEX diffractometer	4873 independent reflections
Radiation source: fine-focus sealed tube	4034 reflections with *I* > 2σ(*I*)
graphite	*R*_int_ = 0.046
ω scans	θ_max_ = 27.5°, θ_min_ = 1.2°
Absorption correction: multi-scan (*SADABS*; Sheldrick, 1996)	*h* = −21→21
*T*_min_ = 0.590, *T*_max_ = 0.828	*k* = −9→9
19142 measured reflections	*l* = −22→22

### Refinement

**Table:**

Refinement on *F*^2^	Primary atom site location: structure-invariant direct methods
Least-squares matrix: full	Secondary atom site location: difference Fourier map
*R*[*F*^2^ > 2σ(*F*^2^)] = 0.053	Hydrogen site location: inferred from neighbouring sites
*wR*(*F*^2^) = 0.156	H-atom parameters constrained
*S* = 1.08	*w* = 1/[σ^2^(*F*_o_^2^) + (0.0837*P*)^2^ + 3.6326*P*] where *P* = (*F*_o_^2^ + 2*F*_c_^2^)/3
4873 reflections	(Δ/σ)_max_ = 0.001
255 parameters	Δρ_max_ = 2.31 e Å^−^^3^
0 restraints	Δρ_min_ = −0.67 e Å^−^^3^

### Fractional atomic coordinates and isotropic or equivalent isotropic displacement parameters (Å^2^)

**Table:**

	*x*	*y*	*z*	*U*_iso_*/*U*_eq_	
Br1	0.25611 (3)	1.02341 (5)	0.45239 (2)	0.03930 (16)	
O1	0.29800 (12)	0.6088 (3)	0.49013 (12)	0.0216 (4)	
O2	0.09230 (13)	0.3244 (3)	0.36320 (12)	0.0240 (5)	
O3	0.11434 (15)	0.5554 (4)	0.28251 (13)	0.0297 (5)	
O4	0.06942 (14)	0.8094 (3)	0.54175 (13)	0.0289 (5)	
O5	0.02390 (13)	0.6629 (3)	0.43103 (13)	0.0259 (5)	
N1	0.30562 (15)	0.4537 (4)	0.37308 (15)	0.0207 (5)	
C1	0.23476 (17)	0.6816 (4)	0.53027 (16)	0.0186 (6)	
C2	0.16009 (17)	0.6470 (4)	0.47364 (16)	0.0185 (6)	
C3	0.17954 (17)	0.5531 (4)	0.41267 (16)	0.0183 (6)	
C4	0.26637 (18)	0.5307 (4)	0.42102 (16)	0.0185 (6)	
C5	0.25368 (19)	0.8840 (4)	0.54633 (17)	0.0240 (6)	
H5A	0.3062	0.8943	0.5787	0.029*	
H5B	0.2128	0.9369	0.5759	0.029*	
C6	0.23270 (17)	0.5743 (4)	0.60559 (16)	0.0190 (6)	
C7	0.25969 (18)	0.3940 (4)	0.61047 (17)	0.0219 (6)	
H7	0.2790	0.3382	0.5672	0.026*	
C8	0.25852 (19)	0.2952 (4)	0.67858 (19)	0.0251 (6)	
H8	0.2771	0.1720	0.6815	0.030*	
C9	0.2307 (2)	0.3741 (5)	0.74210 (19)	0.0281 (7)	
H9	0.2305	0.3060	0.7886	0.034*	
C10	0.2030 (2)	0.5530 (5)	0.73749 (19)	0.0308 (7)	
H10	0.1836	0.6080	0.7809	0.037*	
C11	0.20363 (19)	0.6531 (5)	0.66923 (18)	0.0252 (6)	
H11	0.1841	0.7755	0.6662	0.030*	
C12	0.08049 (18)	0.7155 (4)	0.48724 (17)	0.0212 (6)	
C13	−0.05632 (18)	0.7330 (5)	0.4356 (2)	0.0291 (7)	
H13A	−0.0942	0.6741	0.3957	0.044*	
H13B	−0.0571	0.8660	0.4273	0.044*	
H13C	−0.0717	0.7059	0.4868	0.044*	
C14	0.12496 (18)	0.4812 (4)	0.34460 (17)	0.0194 (6)	
C15	0.0330 (2)	0.2459 (5)	0.3044 (2)	0.0329 (8)	
H15A	0.0136	0.1294	0.3232	0.049*	
H15B	0.0572	0.2240	0.2570	0.049*	
H15C	−0.0120	0.3312	0.2934	0.049*	
C16	0.39323 (18)	0.4490 (5)	0.38899 (18)	0.0236 (6)	
H16	0.4097	0.4544	0.4462	0.028*	
C17	0.4273 (2)	0.6150 (5)	0.3507 (2)	0.0330 (8)	
H17A	0.4051	0.6189	0.2953	0.040*	
H17B	0.4109	0.7288	0.3755	0.040*	
C18	0.5186 (2)	0.6070 (6)	0.3579 (2)	0.0382 (9)	
H18A	0.5385	0.7119	0.3297	0.046*	
H18B	0.5410	0.6176	0.4130	0.046*	
C19	0.5467 (2)	0.4285 (7)	0.3250 (2)	0.0402 (9)	
H19A	0.5280	0.4226	0.2688	0.048*	
H19B	0.6059	0.4249	0.3321	0.048*	
C20	0.5149 (2)	0.2658 (6)	0.3648 (2)	0.0405 (9)	
H20A	0.5370	0.2663	0.4202	0.049*	
H20B	0.5323	0.1512	0.3414	0.049*	
C21	0.4232 (2)	0.2705 (5)	0.3573 (2)	0.0338 (8)	
H21A	0.4010	0.2575	0.3022	0.041*	
H21B	0.4040	0.1656	0.3860	0.041*	

### Atomic displacement parameters (Å^2^)

**Table:**

	*U*^11^	*U*^22^	*U*^33^	*U*^12^	*U*^13^	*U*^23^
Br1	0.0740 (3)	0.0243 (2)	0.0196 (2)	−0.01265 (16)	0.00615 (16)	0.00297 (13)
O1	0.0240 (10)	0.0273 (12)	0.0134 (10)	−0.0023 (8)	0.0023 (8)	−0.0049 (8)
O2	0.0316 (11)	0.0228 (11)	0.0167 (10)	−0.0060 (9)	−0.0010 (8)	−0.0029 (8)
O3	0.0384 (13)	0.0344 (13)	0.0146 (11)	−0.0051 (10)	−0.0029 (9)	0.0058 (9)
O4	0.0321 (12)	0.0330 (13)	0.0214 (11)	0.0071 (10)	0.0022 (9)	−0.0057 (10)
O5	0.0228 (10)	0.0296 (12)	0.0245 (11)	0.0021 (9)	0.0007 (8)	−0.0072 (9)
N1	0.0246 (13)	0.0224 (13)	0.0157 (12)	−0.0007 (10)	0.0047 (9)	−0.0015 (10)
C1	0.0241 (14)	0.0189 (14)	0.0129 (13)	−0.0010 (10)	0.0033 (10)	−0.0019 (10)
C2	0.0261 (14)	0.0156 (13)	0.0132 (12)	−0.0013 (11)	0.0006 (10)	0.0021 (10)
C3	0.0244 (14)	0.0189 (14)	0.0115 (13)	−0.0008 (11)	0.0015 (10)	0.0005 (10)
C4	0.0260 (14)	0.0190 (14)	0.0100 (13)	−0.0037 (11)	0.0007 (10)	−0.0002 (10)
C5	0.0340 (16)	0.0218 (15)	0.0161 (14)	−0.0043 (12)	0.0027 (12)	−0.0008 (11)
C6	0.0209 (13)	0.0217 (14)	0.0139 (13)	−0.0006 (11)	0.0004 (10)	−0.0009 (11)
C7	0.0258 (14)	0.0210 (15)	0.0184 (14)	−0.0001 (11)	0.0007 (11)	−0.0031 (11)
C8	0.0304 (16)	0.0195 (14)	0.0241 (15)	0.0003 (12)	−0.0013 (12)	0.0023 (12)
C9	0.0359 (17)	0.0299 (17)	0.0178 (14)	−0.0010 (13)	0.0009 (12)	0.0061 (13)
C10	0.045 (2)	0.0323 (18)	0.0158 (15)	0.0074 (15)	0.0073 (13)	0.0037 (13)
C11	0.0366 (17)	0.0228 (15)	0.0167 (14)	0.0068 (13)	0.0046 (12)	0.0001 (12)
C12	0.0279 (15)	0.0191 (14)	0.0164 (13)	0.0015 (11)	0.0025 (11)	0.0027 (11)
C13	0.0236 (15)	0.0311 (17)	0.0322 (17)	0.0024 (13)	0.0016 (12)	0.0002 (14)
C14	0.0225 (14)	0.0216 (14)	0.0141 (13)	0.0012 (11)	0.0026 (10)	−0.0018 (11)
C15	0.0348 (18)	0.0362 (19)	0.0265 (17)	−0.0122 (15)	−0.0015 (13)	−0.0112 (14)
C16	0.0241 (15)	0.0324 (17)	0.0141 (14)	−0.0019 (12)	0.0021 (11)	−0.0023 (12)
C17	0.0313 (17)	0.036 (2)	0.0314 (18)	−0.0065 (14)	0.0037 (13)	0.0027 (15)
C18	0.0310 (18)	0.048 (2)	0.036 (2)	−0.0099 (16)	0.0038 (14)	0.0017 (17)
C19	0.0253 (16)	0.063 (3)	0.0332 (19)	−0.0053 (17)	0.0070 (14)	−0.0088 (19)
C20	0.0305 (18)	0.046 (2)	0.044 (2)	0.0075 (16)	0.0026 (15)	−0.0067 (18)
C21	0.0279 (17)	0.0329 (19)	0.041 (2)	0.0002 (14)	0.0073 (14)	−0.0066 (16)

### Geometric parameters (Å, °)

**Table:**

Br1—C5	1.930 (3)	C9—H9	0.9500
O1—C4	1.376 (3)	C10—C11	1.396 (4)
O1—C1	1.448 (3)	C10—H10	0.9500
O2—C14	1.326 (4)	C11—H11	0.9500
O2—C15	1.454 (4)	C13—H13A	0.9800
O3—C14	1.202 (4)	C13—H13B	0.9800
O4—C12	1.204 (4)	C13—H13C	0.9800
O5—C12	1.333 (4)	C15—H15A	0.9800
O5—C13	1.457 (4)	C15—H15B	0.9800
N1—C4	1.261 (4)	C15—H15C	0.9800
N1—C16	1.468 (4)	C16—C21	1.524 (5)
C1—C2	1.518 (4)	C16—C17	1.528 (5)
C1—C5	1.528 (4)	C16—H16	1.0000
C1—C6	1.531 (4)	C17—C18	1.529 (5)
C2—C3	1.339 (4)	C17—H17A	0.9900
C2—C12	1.479 (4)	C17—H17B	0.9900
C3—C4	1.462 (4)	C18—C19	1.521 (6)
C3—C14	1.499 (4)	C18—H18A	0.9900
C5—H5A	0.9900	C18—H18B	0.9900
C5—H5B	0.9900	C19—C20	1.506 (6)
C6—C11	1.392 (4)	C19—H19A	0.9900
C6—C7	1.389 (4)	C19—H19B	0.9900
C7—C8	1.390 (4)	C20—C21	1.535 (5)
C7—H7	0.9500	C20—H20A	0.9900
C8—C9	1.381 (5)	C20—H20B	0.9900
C8—H8	0.9500	C21—H21A	0.9900
C9—C10	1.384 (5)	C21—H21B	0.9900
			
C4—O1—C1	110.3 (2)	O5—C13—H13C	109.5
C14—O2—C15	115.9 (3)	H13A—C13—H13C	109.5
C12—O5—C13	116.2 (2)	H13B—C13—H13C	109.5
C4—N1—C16	119.1 (3)	O3—C14—O2	126.0 (3)
O1—C1—C2	103.1 (2)	O3—C14—C3	124.1 (3)
O1—C1—C5	107.0 (2)	O2—C14—C3	109.9 (2)
C2—C1—C5	114.6 (2)	O2—C15—H15A	109.5
O1—C1—C6	109.0 (2)	O2—C15—H15B	109.5
C2—C1—C6	111.4 (2)	H15A—C15—H15B	109.5
C5—C1—C6	111.3 (2)	O2—C15—H15C	109.5
C3—C2—C12	128.2 (3)	H15A—C15—H15C	109.5
C3—C2—C1	109.5 (3)	H15B—C15—H15C	109.5
C12—C2—C1	122.3 (3)	N1—C16—C21	108.9 (3)
C2—C3—C4	108.8 (2)	N1—C16—C17	108.9 (3)
C2—C3—C14	128.2 (3)	C21—C16—C17	111.0 (3)
C4—C3—C14	123.1 (3)	N1—C16—H16	109.3
N1—C4—O1	125.8 (3)	C21—C16—H16	109.3
N1—C4—C3	126.0 (3)	C17—C16—H16	109.3
O1—C4—C3	108.2 (2)	C18—C17—C16	111.4 (3)
C1—C5—Br1	112.3 (2)	C18—C17—H17A	109.3
C1—C5—H5A	109.1	C16—C17—H17A	109.3
Br1—C5—H5A	109.1	C18—C17—H17B	109.3
C1—C5—H5B	109.1	C16—C17—H17B	109.3
Br1—C5—H5B	109.1	H17A—C17—H17B	108.0
H5A—C5—H5B	107.9	C19—C18—C17	111.2 (3)
C11—C6—C7	119.2 (3)	C19—C18—H18A	109.4
C11—C6—C1	121.3 (3)	C17—C18—H18A	109.4
C7—C6—C1	119.5 (3)	C19—C18—H18B	109.4
C6—C7—C8	120.1 (3)	C17—C18—H18B	109.4
C6—C7—H7	119.9	H18A—C18—H18B	108.0
C8—C7—H7	119.9	C20—C19—C18	110.8 (3)
C9—C8—C7	120.7 (3)	C20—C19—H19A	109.5
C9—C8—H8	119.6	C18—C19—H19A	109.5
C7—C8—H8	119.6	C20—C19—H19B	109.5
C8—C9—C10	119.5 (3)	C18—C19—H19B	109.5
C8—C9—H9	120.3	H19A—C19—H19B	108.1
C10—C9—H9	120.3	C19—C20—C21	110.9 (3)
C9—C10—C11	120.2 (3)	C19—C20—H20A	109.5
C9—C10—H10	119.9	C21—C20—H20A	109.5
C11—C10—H10	119.9	C19—C20—H20B	109.5
C6—C11—C10	120.3 (3)	C21—C20—H20B	109.5
C6—C11—H11	119.9	H20A—C20—H20B	108.0
C10—C11—H11	119.9	C16—C21—C20	111.4 (3)
O4—C12—O5	125.0 (3)	C16—C21—H21A	109.3
O4—C12—C2	123.6 (3)	C20—C21—H21A	109.3
O5—C12—C2	111.4 (3)	C16—C21—H21B	109.3
O5—C13—H13A	109.5	C20—C21—H21B	109.3
O5—C13—H13B	109.5	H21A—C21—H21B	108.0
H13A—C13—H13B	109.5		
			
C4—O1—C1—C2	−3.2 (3)	C11—C6—C7—C8	−0.8 (4)
C4—O1—C1—C5	−124.4 (2)	C1—C6—C7—C8	179.7 (3)
C4—O1—C1—C6	115.1 (2)	C6—C7—C8—C9	0.0 (5)
O1—C1—C2—C3	4.5 (3)	C7—C8—C9—C10	0.6 (5)
C5—C1—C2—C3	120.4 (3)	C8—C9—C10—C11	−0.3 (5)
C6—C1—C2—C3	−112.2 (3)	C7—C6—C11—C10	1.1 (5)
O1—C1—C2—C12	−174.1 (2)	C1—C6—C11—C10	−179.4 (3)
C5—C1—C2—C12	−58.2 (4)	C9—C10—C11—C6	−0.6 (5)
C6—C1—C2—C12	69.2 (3)	C13—O5—C12—O4	2.8 (5)
C12—C2—C3—C4	174.5 (3)	C13—O5—C12—C2	−176.0 (3)
C1—C2—C3—C4	−4.0 (3)	C3—C2—C12—O4	−175.0 (3)
C12—C2—C3—C14	−4.6 (5)	C1—C2—C12—O4	3.3 (5)
C1—C2—C3—C14	176.9 (3)	C3—C2—C12—O5	3.8 (4)
C16—N1—C4—O1	−2.1 (5)	C1—C2—C12—O5	−177.9 (3)
C16—N1—C4—C3	177.6 (3)	C15—O2—C14—O3	−5.8 (4)
C1—O1—C4—N1	−179.2 (3)	C15—O2—C14—C3	175.4 (3)
C1—O1—C4—C3	1.1 (3)	C2—C3—C14—O3	100.1 (4)
C2—C3—C4—N1	−177.8 (3)	C4—C3—C14—O3	−78.9 (4)
C14—C3—C4—N1	1.4 (5)	C2—C3—C14—O2	−81.1 (4)
C2—C3—C4—O1	2.0 (3)	C4—C3—C14—O2	99.9 (3)
C14—C3—C4—O1	−178.9 (3)	C4—N1—C16—C21	146.3 (3)
O1—C1—C5—Br1	61.4 (3)	C4—N1—C16—C17	−92.6 (3)
C2—C1—C5—Br1	−52.2 (3)	N1—C16—C17—C18	−173.9 (3)
C6—C1—C5—Br1	−179.67 (19)	C21—C16—C17—C18	−54.1 (4)
O1—C1—C6—C11	153.4 (3)	C16—C17—C18—C19	55.4 (4)
C2—C1—C6—C11	−93.6 (3)	C17—C18—C19—C20	−57.0 (4)
C5—C1—C6—C11	35.6 (4)	C18—C19—C20—C21	57.2 (4)
O1—C1—C6—C7	−27.2 (3)	N1—C16—C21—C20	174.2 (3)
C2—C1—C6—C7	85.9 (3)	C17—C16—C21—C20	54.4 (4)
C5—C1—C6—C7	−145.0 (3)	C19—C20—C21—C16	−56.3 (4)
